# Can hyperbaric oxygen be used to prevent deep infections in neuro-muscular scoliosis surgery?

**DOI:** 10.1186/1471-2482-14-85

**Published:** 2014-10-27

**Authors:** Mustafa Erkan Inanmaz, Kamil Cagri Kose, Cengiz Isik, Halil Atmaca, Hakan Basar

**Affiliations:** Department of Orthopedics and Traumatology, Sakarya University Faculty of Medicine, Sakarya Universitesi Araştırma Hastanesi Ortopedi ve Travmatoloji Kliniği, Sakarya, Turkey; Department of Orthopedics and Traumatology, Marmara University Faculty of Medicine, Istanbul, Turkey; Department of Orthopedics and Traumatology, Abant Izzet Baysal University Faculty of Medicine, Bolu Merkez/Bolu, Turkey; Department of Orthopedics and Traumatology, Akdeniz University Faculty of Medicine, Antalya, Turkey

**Keywords:** Neuromuscular scoliosis, Infection, Treatment, Hyperbaric oxygen

## Abstract

**Background:**

The prevalence of postoperative wound infection in patients with neuromuscular scoliosis surgery is significantly higher than that in patients with other spinal surgery. Hyperbaric oxygen has been used as a supplement to treat postsurgical infections. Our aim was to determine beneficiary effects of hyperbaric oxygen treatment in terms of prevention of postoperative deep infection in this specific group of patients in a retrospective study.

**Methods:**

Forty two neuromuscular scoliosis cases, operated between 2006–2011 were retrospectively reviewed. Patients who had presence of scoliosis and/or kyphosis in addition to cerebral palsy or myelomeningocele, postoperative follow-up >1 year and posterior only surgery were the subjects of this study. Eighteen patients formed the Hyperbaric oxygen prophylaxis (P-HBO) group and 24, the control group. The P-HBO group received 30 sessions of HBO and standard antibiotic prophylaxis postoperative, and the control group (received standard antibiotic prophylaxis).

**Results:**

In the P-HBO group of 18 patients, the etiology was cerebral palsy in 13 and myelomeningocele in 5 cases with a mean age of 16.7 (11–27 yrs). The average follow-up was 20.4 months (12–36mo). The etiology of patients in the control group was cerebral palsy in 17, and myelomeningocele in 7 cases. The average age was 15.3 years (8–32 yrs). The average follow-up was 38.7 months (18–66mo). The overall incidence of infection in the whole study group was 11.9% (5/42). The infection rate in the P-HBO and the control group were 5.5% (1/18), and 16.6% (4/24) respectively. The use of HBO was found to significantly decrease the incidence of postoperative infections in neuromuscular scoliosis patients.

**Conclusion:**

In this study we found that hyperbaric oxygen has a possibility to reduce the rate of post-surgical deep infections in complex spine deformity in high risk neuromuscular patients.

## Background

The prevalence of postoperative wound infection after scoliosis surgery is significantly higher in neuromuscular scoliosis patients when compared to other types of scoliosis [1]. It may also compromise the correction obtained by surgery, especially in cases in which hardware removal is necessary. There are various factors that may contribute to the increased risk for postoperative wound infections in patients with neuromuscular disease. Severe neurologic involvement, a diagnosis of myelodysplasia, the use of allograft bone, preoperative malnutrition, urinary tract infection, and excessive blood loss have all been identified as potential risk factors [1–3].

Hyperbaric oxygen (HBO) has been reported to heal postoperative spinal infections in adults with intact osteosynthesis material [4]. HBO therapy increases oxygen tension in tissues, including bone, with increased bone-turnover and increased bone-metal contact [5].

The purpose of this retrospective study was to compare and evaluate the infection rate of neuromuscular scoliosis patients undergoing instrumented only-posterior spinal fusion who were treated with or without early postoperative prophylactic administration of HBO (P-HBO). We hypothesized that the use of hyperbaric oxygen in addition to standard prophylaxis would result in a lower infection rate without adverse clinical effects.

## Methods

This study was done at a university hospital. Since April 2009 patients with neuromuscular spinal deformities had received HB0 postoperative with the aim of reducing the incidence of postoperative infections and wound healing problems. To determine the effect of supplemental HBO on the infection rates in our patients, the neuromuscular deformity cases, operated between 2006–2011, were retrospectively reviewed. A total of 55 patients with neuromuscular deformities were operated within the given time period. The inclusion criteria were: presence of scoliosis and/or kyphosis in addition to cerebral palsy or myelomeningocele, postoperative follow-up >1 year and posterior only surgery. Patients who did not meet these criteria, or who did not have enough data in the patient file were excluded.

We had a total of 42 patients meeting these inclusion criteria. The reasons for exclusion of 13 cases were: presence of a neuromuscular condition other than the above [6], anterior/posterior combined surgery [3] and insufficient data [4]. Eighteen patients formed the P-HBO group and 24 the control group. The patients and/or their families were informed that their data would be submitted for publication, and gave their informed consent which was archived in the patients’ folders. The study was approved by the local ethics committee of Sakarya University.

Preoperative risk factors for infection were: prior spine surgery, degree of cognitive delay, nutritional parameters including albumin and total lymphocyte count, and prior urinary tract infection and decubiti. Operative time, estimated blood loss (EBL), and number of levels fused were obtained from the chart. Finally, we evaluated the postoperative clinical parameters: infection rate, time of infection, implant removal due to infection, re-operations, pseudoarthrosis. The primary outcome evaluated was the incidence of infection.

Deep wound infection for the purpose of this study, and as described in the literature is defined as a surgical site infection in which there is a communication between the associated infected material and the spinal instrumentation and bone graft/fusion mass, as proven by surgical exploration. The types of prophylactic antibiotics used and the duration of their administration were the same in both groups. All patients received standard systemic antibiotic prophylaxis consisting of 1 g IV Cefazolin 1 hour before surgical incision and this was followed by 1 g IV Cefazolin every 8 hours for 3 days. Patients in both groups underwent the same intraoperative technique by the same surgeon and the same postoperative protocol. After surgery, patients in both groups were managed in the same manner the only difference being is the application of prophylactic HBO in one group starting after surgery.

All patients in the HBO group were treated in a multiplace hyperbaric chamber (BarOtechNEO; BarOtech®INC), Hyperbaric (100%) oxygen was administered at a pressure of 2.4 ATA for 90 minutes/day. Patients underwent 30 sessions of treatment over a 6 weeks period (5 sessions/week).

## Results

Forty-two patients were enrolled in the study. The mean age at the time of surgery was 15.7 years (8–32 yrs). The average follow-up after the index procedure was 2.3 years. Thirty patients had cerebral palsy and 12 had myelomeningocele.

In the P-HBO group of 18 patients, the etiology was cerebral palsy in 13 and myelomeningocele in 5 cases with a mean age of 16.7 (11–27 yrs). The average follow-up was 20.4 months (12–36 mo). This group was routinely administered HBO starting on postoperative second or third day. The control group consisted of 24 patients and the etiology was cerebral palsy in 17, myelomeningocele in 7 cases. The average age was 15.3 years (8–32 yrs). The average follow-up was 38.7 months (18–66 mo).

One patient in each group with a stiff and severe deformity had undergone preoperative halo traction for 2 weeks. In 7 patients, spinal fixation was extended down to the pelvis using iliac screws. Posterior correction was performed using pedicular screws, intralaminar screws, laminar hooks, sublaminar wires and rods. Only titanium implants (Tasarım Med®INC, Istanbul, Turkey) were used for the spinal correction. At the end of the procedure, a mixture of autograft (harvested on spinous processes) and allograft cancellous chips (Allograft Innovations, LLC Gainsville, FL, USA) were placed to enhance bony fusion.

There were no significant differences between the HBO group and the control group in terms of the number of fused levels, the amount of intraoperative bleeding, and the duration of operation (Table 1).

**Figure 1:**
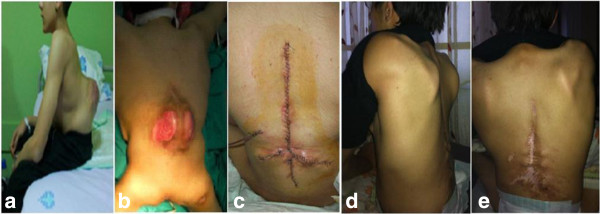
**A-14-year-old male patient with congenital kyphosis due to meningomyelocele. a**: lateral and **b**: posterior view of the patient showing infected skin ulcers at the apex of the deformity and patient was predicted as high risk for infection **c**: The wound at postoperative day 1. Prophylactic HBO was started after surgery. **d**: Lateral and **e**: posterior clinical presentation of patient at postoperative 24th month. Written informed consent was obtained from the patient for publication of Figure 1 images.

One patient in the P-HBO group had mild mental retardation, as compared with two in the control group. Four patients in the P-HBO and five in the control group had prior back surgery (closure of meningomyelocele in the newborn period). There were insufficient data available to compare nutrition markers and previous urinary tract infections. Three meningocele patients had decubiti on the surgical area (Two in the P-HBO (Figure 1) and one in the control group).

**Table 1:** **Details of unpaired** ***t*** **test comparison of mean study control and P-HBO groups SD: standart deviation**

	Groups	N	Mean	SD	t value	***P***
FUSED LEVELS (no of vertebra)	P-HBO	18	13,2	2,84	-0,613	0,544
	Control	24	12,7	2,40		
BLOOD LOSS (ml)	P-HBO	18	1370	352	-0,614	0,543
	Control	24	1280	530		
SURGERY TIME (min)	P-HBO	18	244,8	53,1	-0,032	0,974
	Control	24	244,3	47,6

There was an overall incidence of 11.9% (5/42) of infection in the whole study group. The rate of infection was altered by the underlying diagnosis. The incidence of infection among the patients with cerebral palsy was 10% (3/30) and the incidence of infection among the patients with myelomeningocele was 16.6% (2/12). The infection rate in the P-HBO and the control group were 5.5% (1/18), and 16.6% (4/24) respectively. When the infection rates were compared according to the primary diagnosis, none of the myelomeningocele patients had infection in the P-HBO group compared to 2 (28.5%) in the control group. The infection rates in cerebral palsy patients were 7.6% (1/13) and 11.7% (2/17) in the P-HBO and the control groups respectively (Table 2).

**Table 2:** **Group-specific infection rates CP: cerebral palsy, MM: myelomeningocele, Inf: infection**

	Total			CP			MM		
	#	Inf	Rate	#	Inf	Rate	#	Inf	Rate
P-HBO	18	1	%5,5	13	1	%7,6	5	0	%0
Control	24	4	%16,6	17	2	%11,7	7	2	%28,5
Overall	42	5	%11,9	30	3	%10	12	2	%16,6

Complications were evaluated in terms of reoperations, implant removal and pseudarthrosis. The average number of reoperations was 2.8/infected patient. All patients received antibiotics according to their bacterial cultures as advised by specialists in infectious diseases. One patient had persistent pseudoarthrosis at the final follow-up. No immediate or delayed adverse effects related to the use of HBO were noted.

## Discussion

Despite the use of prophylactic systemic antibiotics and improved surgical technique, surgical site infections remain a serious concern in neuromuscular patients. The rate of wound infection after surgery for idiopathic scoliosis ranges from 1% to 5%. In contrast, the rate of infection in spinal surgery for deformity related to myelomeningocele has been reported to be from 8% to 24%. The rate of infection in spinal surgery for deformity related to cerebral palsy has been reported to be from 6.1% to 8.7%. [1–3, 6, 7]. The use of prophylactic HBO in neuromuscular deformity patients has been reported twice in the literature. Kose et al. [8] used supplemental HBO in a meningomyelocele patient who underwent three level kyphectomy. Although the skin overlying the kyphotic segment was infected and ulcerated before the operation, the wound healed without any problems postoperatively. Larrson et al. [9] used preoperative HBO-treatment because of the calculated high risk for infection in two patients. Both had suffered postoperative infections at several previous surgical interventions, and did so despite HBO prophylaxis. They were treated with a prolonged HBO protocol, and eventually healed without removal of implants. In our study, the infection rate of 16.6% in the control group is similar to the rates in the literature however; the infection rate of 5.5% in the P-HBO group is significantly lower.

Hyperbaric oxygen therapy (HBOT) has been added to treatment protocols in chronic wound problems with successful outcomes. Dramatic beneficial effects of HBOT were also observed in patients with spinal infections caused by a variety of microorganisms. Ravicovitch and Spallone [10] obtained good results for treatment of spinal epidural abscesses with HBOT after laminectomy. Larsson et al. [4, 9] applied HBOT for the treatment of neurosurgical infections after cranial and spinal surgeries. These investigations have primarily focused on the treatment of infected wounds. Recently, there has been an increasing interest for prophylaxis against infection. For this reason, HBOT has been used as a treatment for various acute wounds. Bouachour et al. [11] studied HBOT in patients with crush injuries where complete wound healing was achieved in 17 patients (94%) treated with HBOT versus 10 patients (56%) in the sham-HBOT group, showing a statistically significant difference in favor of HBOT. Huang et al. [12] studied a prospective, 16 patients with complex open elbow injuries who were treated with a combination of treatment modalities. The treatment protocol also used HBOT to facilitate immediate internal fixation. The results of this study demonstrated that the studied treatment protocol provides a promising alternative for managing these complex open elbow injuries. Neuromuscular patients who lack bowel and bladder control risk seeding a wound with feces or urine and development of decubiti lead to an infection by direct contamination. A persistent cerebrospinal fluid leak after surgery may interfere with wound healing and is a favorable medium for bacterial growth. Poor personal hygiene and soiling of the wound also may be factors [2]. The first few hours after tissue is contaminated by bacteria constitute a critical period during which wound infections are established. Therefore, per operative factors influence the incidence of infections, even though infections are typically not detected until some days after surgery. The factors that influence the incidence of surgical-wound infection include complexity of surgery, the patient’s underlying illness, poor local blood and oxygen supply, or damage to the vasculature. If one of these complicating factors occurs, these wounds can be considered potentially problematic wounds with an extended healing time. In these cases, more specific care is needed [13–15]. Based on these literature data, we thought that per operative HBO support might reduce the incidence of infection and wound healing problems in our patients.

All wounds disrupt the local vascular supply as a result of injury and thrombosis of vessels, which cause wounds to be hypoxic when compared with normal tissue. Resistance to infection therefore, depends on the partial pressure of oxygen in the wound and can thus potentially be improved by increasing the arterial oxygen tension beyond that required to saturate blood. The therapeutic effect of HBO treatment with regard to infections is mainly attributable to reduction of hypoxia in tissues with significant improvement in leukocyte and phagocyte killing capacity [9, 16]. Healing is impaired at low oxygen tensions which are often observed in wounds [17]. Cells such as neutrophils, fibroblasts, macrophages, osteoclasts and osteoblasts are all dependent upon an environment in which oxygen is not deficient in order to carry out their specific anti-infectious, inflammatory or repair functions [16]. Oxygen has for a long time been used in clinical practice to enhance wound healing. Systemic administration of oxygen through the lung and the cardiovascular system, however, is known to improve wound healing and decrease the risk of infection. Hopf et al. used supplemental oxygen in surgical patients and found that supplementary oxygen in the inspired air for the first 2 postoperative days has reduced the postoperative wound infection rate after colorectal surgery. Supplementary oxygen treatment should (from their point of view) be a standard procedure for the treatment of all colorectal patients [13, 18].

Animal experiments using microelectrodes to measure oxygen partial pressures in normal, healing, and infected tissues and in tissues containing foreign material have demonstrated marked hypoxia, especially when infected foreign material was present [19]. HBO therapy can greatly increase the oxygen concentration in all body tissues by hyper oxygenation. It also decreases lesion-tissue edema through vasoconstriction, and resists superimposed infection via an improvement in white blood cell phagocytosis and antibiotic synergy. Thus it was added to immediate internal fixation and early soft-tissue coverage to refine clinical results [9, 13, 20, 21].

The removal of spinal implants at an early postoperative stage can compromise fusion and curve correction. As such, retention of implants should be one of the goals when treating infection. Buchowski et al. [22] reported that 39.1% of patients with spinal deformity and postoperative infections required instrumentation removal. In the study by Ho et al. [23] implant removal was necessary in 10 of the 53 patients with postoperative infections after spinal deformity surgery. The incidence of pseudarthrosis in subjects with an infection was 25% (13 of 51). Larrson et al. [9] reported a consecutive series of six children with neuromuscular spine deformity who received HBO as an adjunct in the management of postoperative deep infection after complex correction surgery. The spinal implants remained intact without any bone healing problems or radiological signs of loosening. In our series, two of five patients who had infection required implant removal. One had infection in addition to implant prominence which resulted in repeated skin breakdown and the other had an infection which persisted despite serial irrigation and debridement. In the remaining 3 patients, HBO was added to the treatment and these recovered without any need for implant removal. These support the results of Larsson et al. [9]. When we analyzed the durations of post infection treatment we found that the 3 patients with HBOT recovered. One of the patients which required implant removal had persistent pseudoarthrosis.

Significant blood loss, use of allograft, increased number of fused levels, lenghtened operation time have all been cited as potential risk factors for infection in patients with neuromuscular scoliosis [1–3, 24]. One of the strong points of our study is that both groups are similar in terms of surgical technique (blood loss, use of allograft, fused levels, operation time) and post operative treatment regimen as all patients were operated by the same surgeon. Elimination of the variability of these infection risk factors further amplifies the effect of HBO treatment.

There are several limitations to this study. The study was limited in that it was conducted in a retrospective fashion. Also, a direct comparison of final outcomes in patients with infection who undergo P-HBO versus who receive standard prophylaxis is complicated by several factors. Even in patients with similar co morbidities, the local and systemic factors that hinder infection cure and wound healing vary greatly between individuals [20]. It is possible that those undergoing HBOT had closer surveillance and more detailed nursing care than those who did not. Finally, the small sample size of this case series makes it difficult to stratify the risk factors in the patients, which would be useful for evaluating the efficacy of HBO prophylaxis.

## Conclusion

This work describes an alternative prophylaxis protocol with promising preliminary results for managing wound care after neuromuscular deformity surgery. HBO is a safe and potentially useful supplement to prevent postoperative deep infections in complex spine deformity in high risk neuromuscular patients. Additional prospective studies are needed to further substantiate the effectiveness of this method in reducing postoperative spinal surgical site infections.

### Consent

Written informed consent was obtained from the patient for publication of Figure 1 images.
